# Kikuchi-Fujimoto Disease With Isolated Mediastinal Lymphadenopathy: A Rare Presentation of an Even Rarer Disease

**DOI:** 10.7759/cureus.72921

**Published:** 2024-11-03

**Authors:** Junaid Zafar Sheikh, Louay Kila, Irfan Amin, Umar Khan, Brian Casserly

**Affiliations:** 1 Respiratory Medicine, University Hospital Limerick, Limerick, IRL

**Keywords:** fine needle aspiration, kikuchi-fujimoto disease, lupus erythematosus, mediastinal lymphadenopathy, necrotizing lymphadenopathy

## Abstract

Kikuchi-Fujimoto disease (KFD) is a rare self-limiting condition presenting as fever and cervical lymphadenopathy, with only two reported cases with isolated mediastinal lymphadenopathy. Lack of awareness about this condition often results in a high rate of misdiagnosis. We present a case of a 29-year-old Indian male with fever, mucocutaneous ulcers, weight loss, and mediastinal lymphadenopathy on CT. Extensive investigations ruled out malignancy and autoimmune and infectious causes. Endobronchial ultrasound-guided fine needle aspiration revealed necrotizing lymphadenitis, indicating KFD, followed by symptomatic management and reassurance. Awareness is crucial to avoid misdiagnosis and unnecessary treatment due to its potentially serious differentials. Long-term follow-up is imperative due to its association with autoimmune conditions.

## Introduction

Kikuchi-Fujimoto disease (KFD), also termed histiocytic necrotizing lymphadenitis, is an enigmatic and rare condition first described independently by Kikuchi and Fujimoto in Japan in 1972. The disease predominantly affects young adults, with a slight female predominance, and is characterized by its self-limiting course [1]. The hallmark clinical features include fever and tender lymphadenopathy, typically localized to the cervical region [2]. However, extranodal manifestations such as skin rash, hepatosplenomegaly, and night sweats can occur, contributing to the disease's diagnostic complexity. Despite its global occurrence, KFD remains under-recognized, which is problematic given the substantial overlap in clinical presentation with more severe conditions such as lymphomas, systemic lupus erythematosus (SLE), and infectious processes like tuberculosis [3].

The pathophysiology of KFD is not well understood but is thought to involve an aberrant immune response to an infectious agent or a direct autoimmune process [4]. This theory is supported by the frequent association of KFD with viral infections and autoimmune markers in some patients. Histologically, KFD is characterized by paracortical areas of necrosis with abundant karyorrhectic debris, typically devoid of neutrophils or granulomas, distinguishing it from other lymphadenopathies [5]. Given the nonspecific symptoms and the rarity of the condition, KFD is frequently misdiagnosed, with some studies indicating misdiagnosis rates as high as 40% [2]. This high rate of misdiagnosis underscores the need for heightened awareness and knowledge of the disease among clinicians to prevent unnecessary and potentially harmful interventions.

## Case presentation

We present the case of a 29-year-old Indian gentleman who arrived at the hospital with a perplexing and progressively worsening set of symptoms. He was troubled by painful mucocutaneous ulcers in his mouth and around his perianal region, alongside a persistent fever, throbbing headaches, and an overwhelming sense of malaise that had persisted for more than three weeks. His condition was further complicated by recurrent episodes of intense chills, unexplained weight loss, and fresh rectal bleeding. Notably, the patient denied any penile discharge, respiratory complaints such as cough or sputum production, urinary symptoms, or recent contact with individuals suffering from infectious diseases like tuberculosis. He had no history of travel, did not smoke, and did not drink alcohol. His past medical history was uneventful, offering no clues to explain his current symptoms.

**Figure 1:**
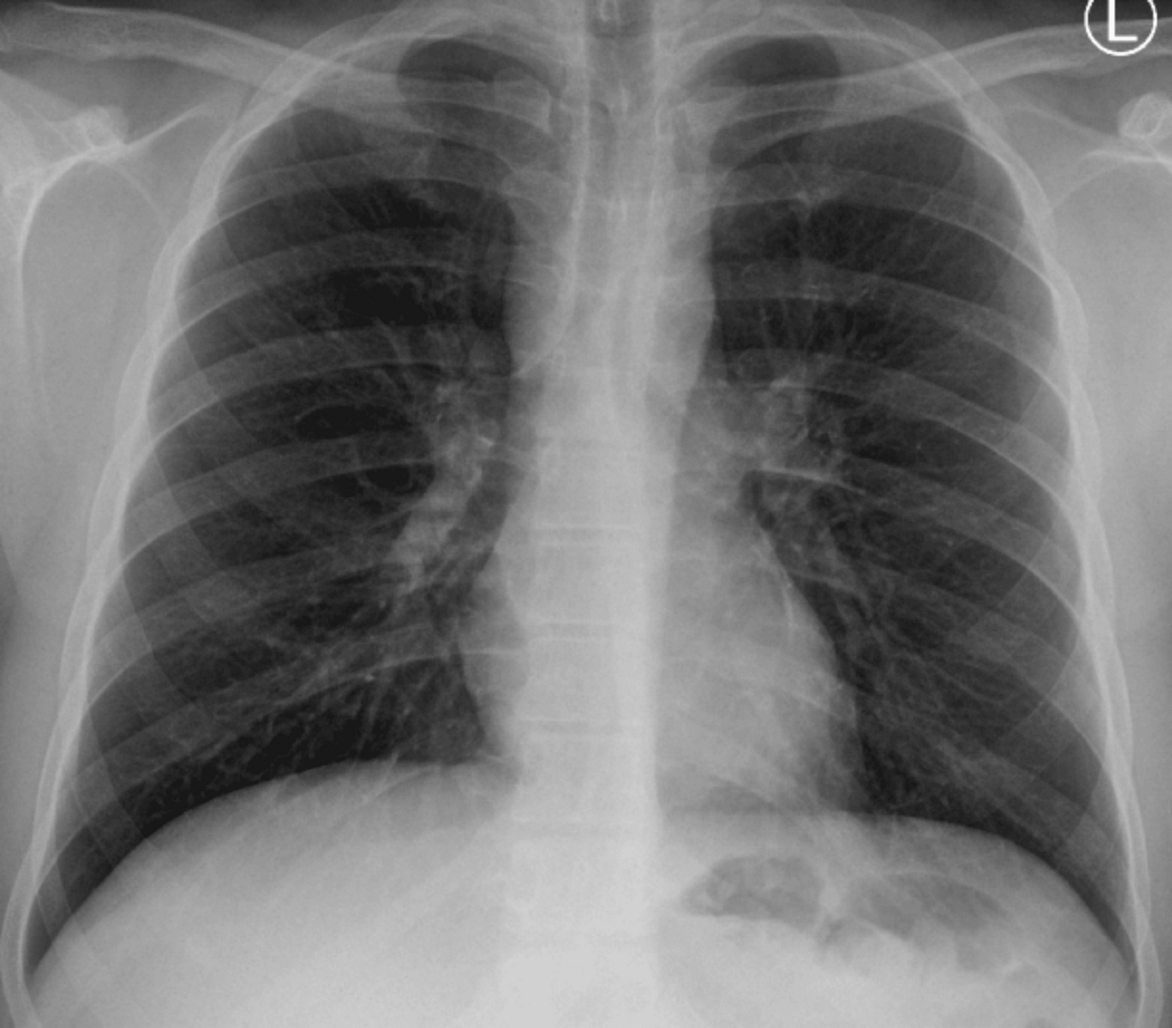
Chest radiograph showing normal appearance of the lungs

Upon his admission, an initial round of investigations revealed elevated inflammatory markers, signaling a potential underlying inflammatory or infectious process. However, despite thorough testing, blood cultures and wound swabs came back negative. Imaging studies, including a chest X-ray and a CT scan of the brain, were similarly unremarkable. With an infectious etiology still high on the list of possibilities, the patient was empirically started on intravenous antibiotics and corticosteroids. Extensive screening for infectious agents and autoimmune conditions followed. Tests for HIV, hepatitis, syphilis, tuberculosis, chlamydia, gonorrhea, herpes simplex virus, varicella-zoster virus, and fungal cultures all returned negative. Although his Indian background placed him at higher risk for tuberculosis, he had no known exposure to individuals with the disease. Given his prolonged low-grade fever, tuberculosis remained a consideration, but a chest X-ray appeared normal (Figure 1), and a QuantiFERON-TB Gold test ruled out latent infection.

Further investigations deepened the mystery. Serum protein electrophoresis, complement levels, and a vasculitic screen yielded normal results. Likewise, tests for SLE, including antinuclear antibodies and anti-dsDNA, were negative. Stool examinations showed no evidence of parasitic or bacterial infection. The dermatological evaluation included a skin punch biopsy of the ulcerative lesions, yet this too failed to provide any diagnostic clues. Screening for Behçet’s disease was considered due to the presence of oral and perianal ulcers, but the absence of the HLA-B51 antigen lessened its likelihood.

Due to his gastrointestinal symptoms, a sigmoidoscopy was performed, revealing no abnormalities, and a terminal ileal biopsy similarly returned negative findings. A colonoscopy was arranged on an outpatient basis but was also unremarkable. The diagnostic picture remained frustratingly unclear.

**Figure 2:**
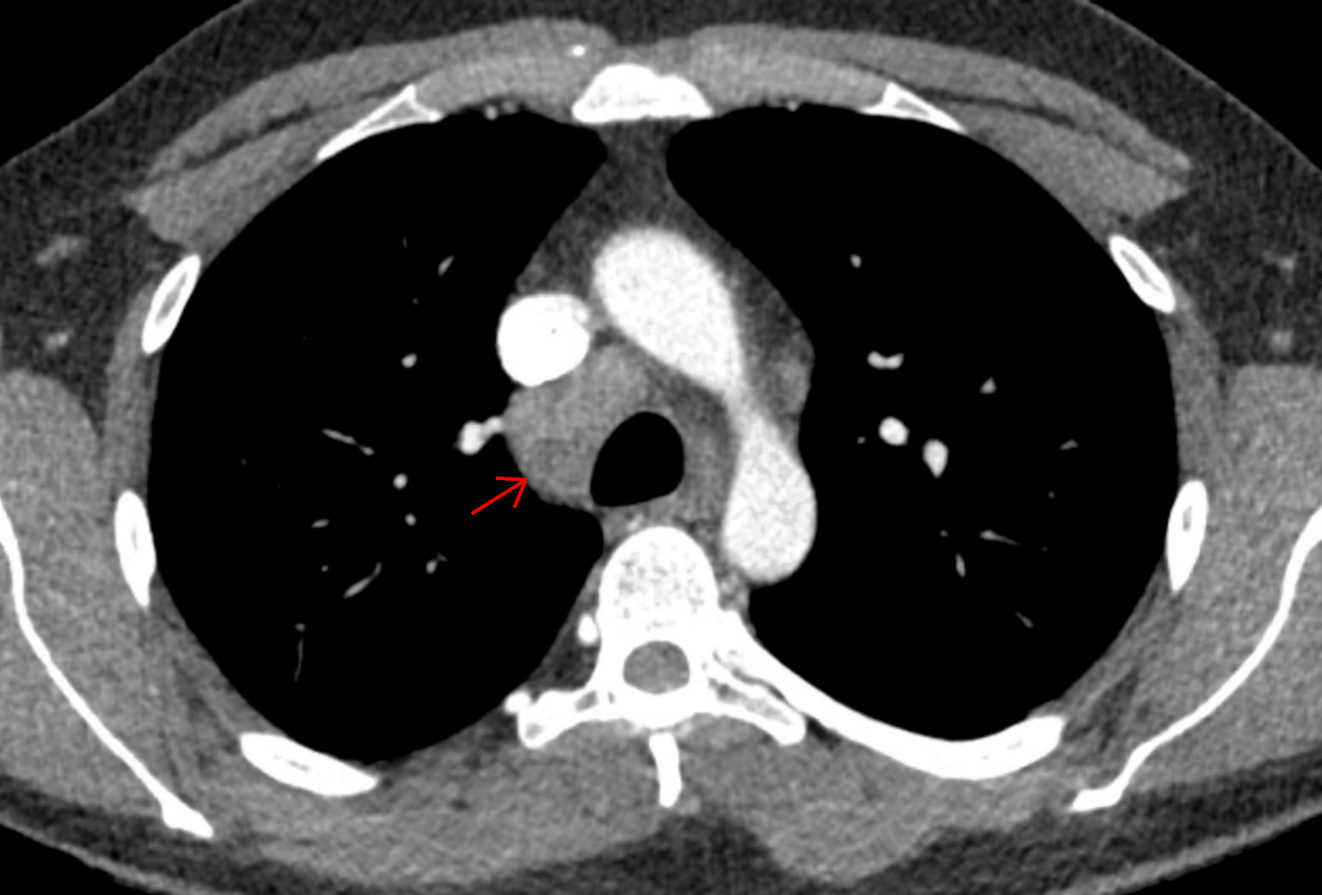
CT thorax showing enlarged right paratracheal lymph node CT: computed tomography

**Figure 3:**
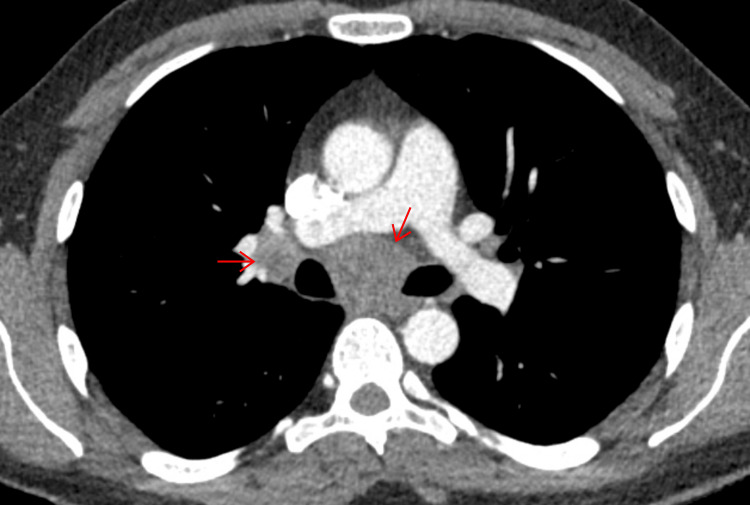
CT thorax showing enlarged subcarinal and right hilar lymphadenopathy CT: computed tomography

A CT scan of the thorax, abdomen, and pelvis offered the first significant clue: mediastinal lymphadenopathy involving the right paratracheal, subcarinal, and right hilar lymph nodes (Figures 2-3).

There were no notable findings in the abdomen or pelvis, but the discovery of enlarged lymph nodes in the chest prompted further action. Both the hematology and respiratory teams were consulted, and it was agreed that a more invasive approach was necessary to obtain a definitive diagnosis. The patient underwent an endobronchial ultrasound (EBUS)-guided fine needle aspiration (FNA) of the affected lymph nodes.

During his hospital stay, the patient remained clinically stable, and his symptoms gradually improved. He was eventually discharged, with a plan for follow-up pending the results of the lymph node biopsy. In subsequent outpatient visits, the patient’s condition continued to improve significantly. He experienced a complete resolution of his symptoms, and the EBUS FNA results revealed benign cytology with extensive necrosis. Stains for tuberculosis were negative, effectively ruling out both malignancy and infectious causes such as tuberculosis. With all infectious and malignant processes ruled out, the most likely diagnosis became KFD, a rare but benign cause of lymphadenopathy.

**Figure 4:**
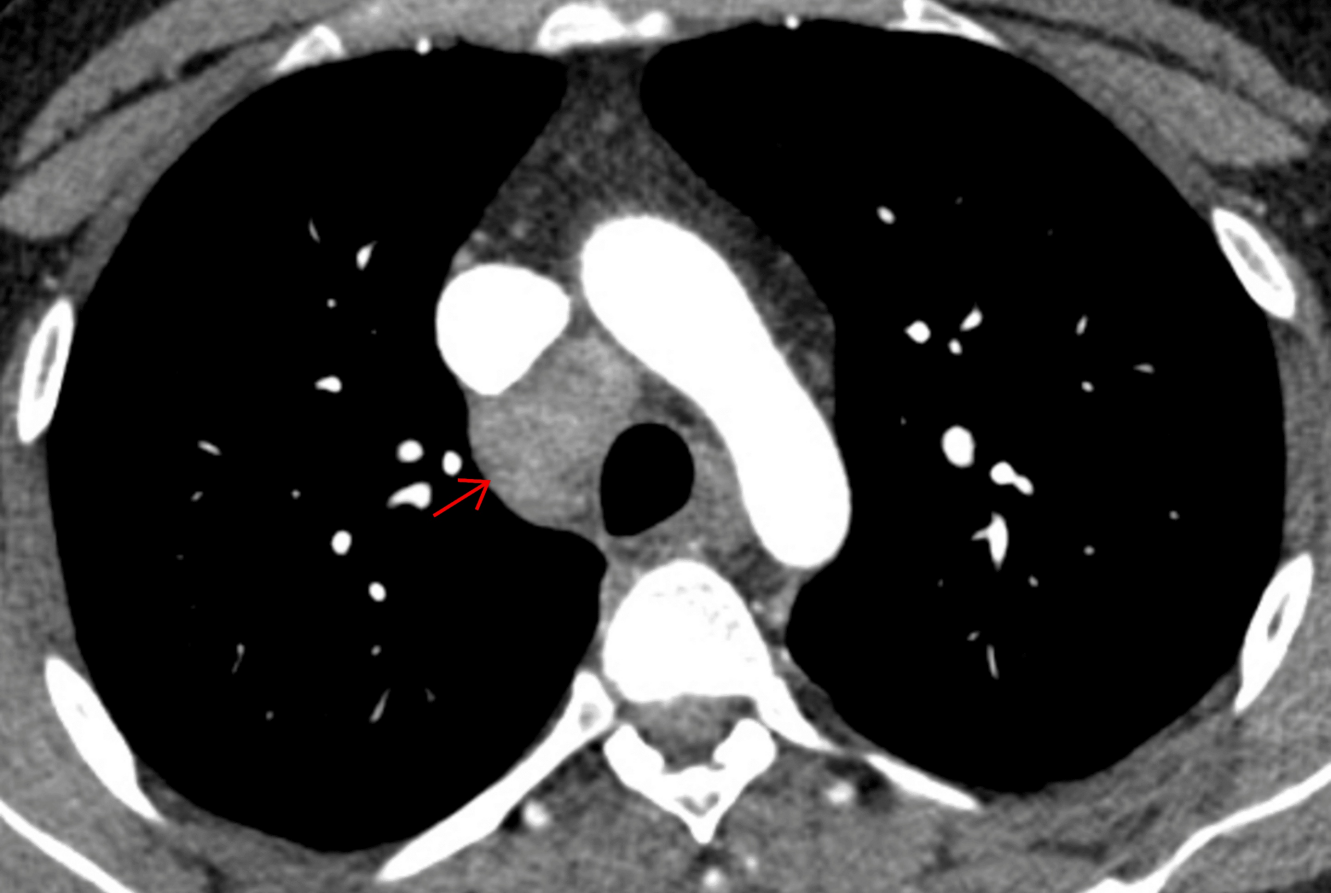
CT thorax showing interval reduction in the size of right paratracheal lymph node CT: computed tomography

**Figure 5:**
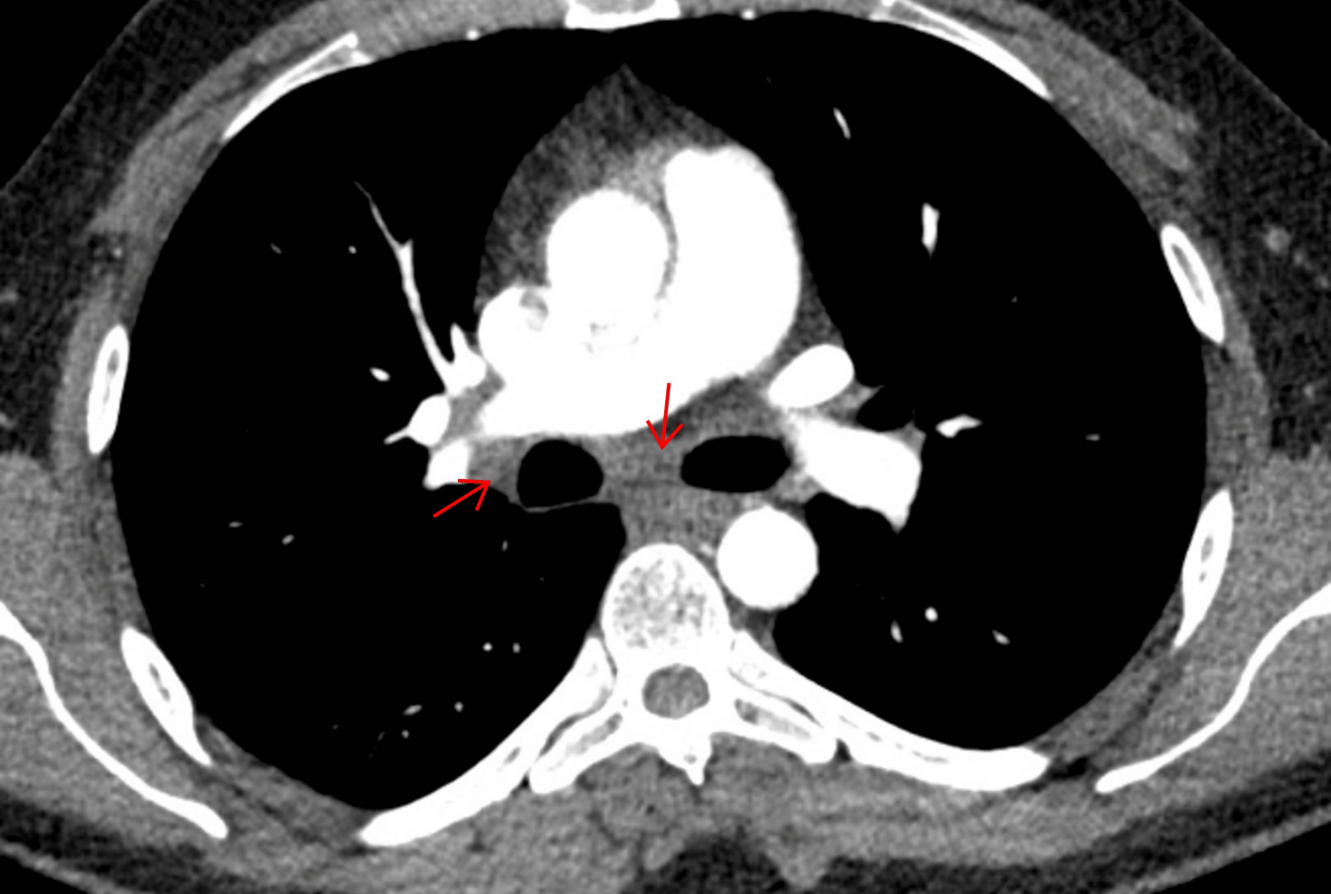
CT thorax showing interval resolution of right hilar and subcarinal lymph node CT: computed tomography

To monitor his recovery, follow-up imaging was performed six months after his initial CT scan. This revealed a remarkable resolution of the right hilar and mediastinal lymphadenopathy, as well as a reduction in the size of the right paratracheal lymph node (Figures 4-5).

**Figure 6:**
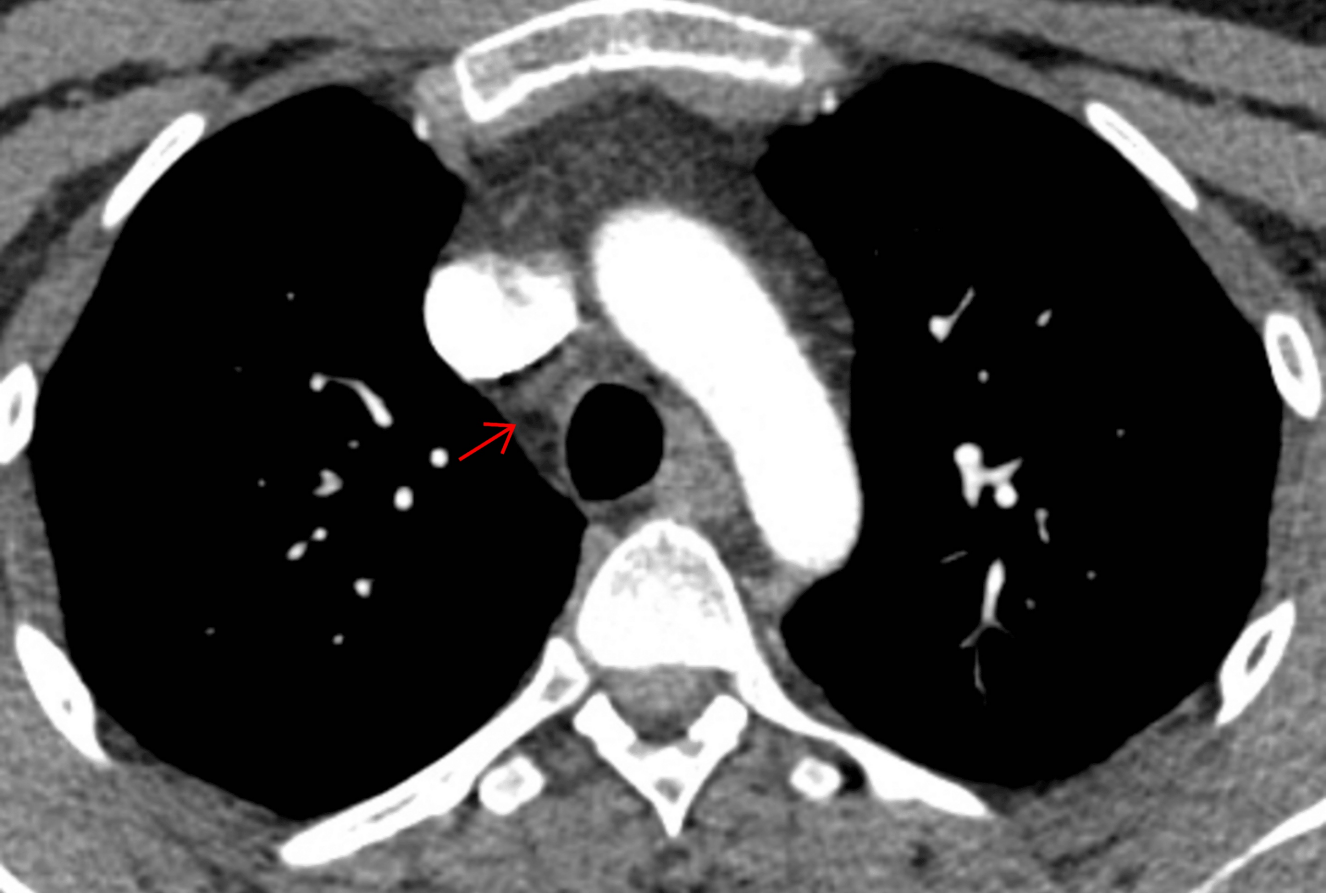
CT thorax showing complete resolution of right paratracheal lymph node CT: computed tomography

**Figure 7:**
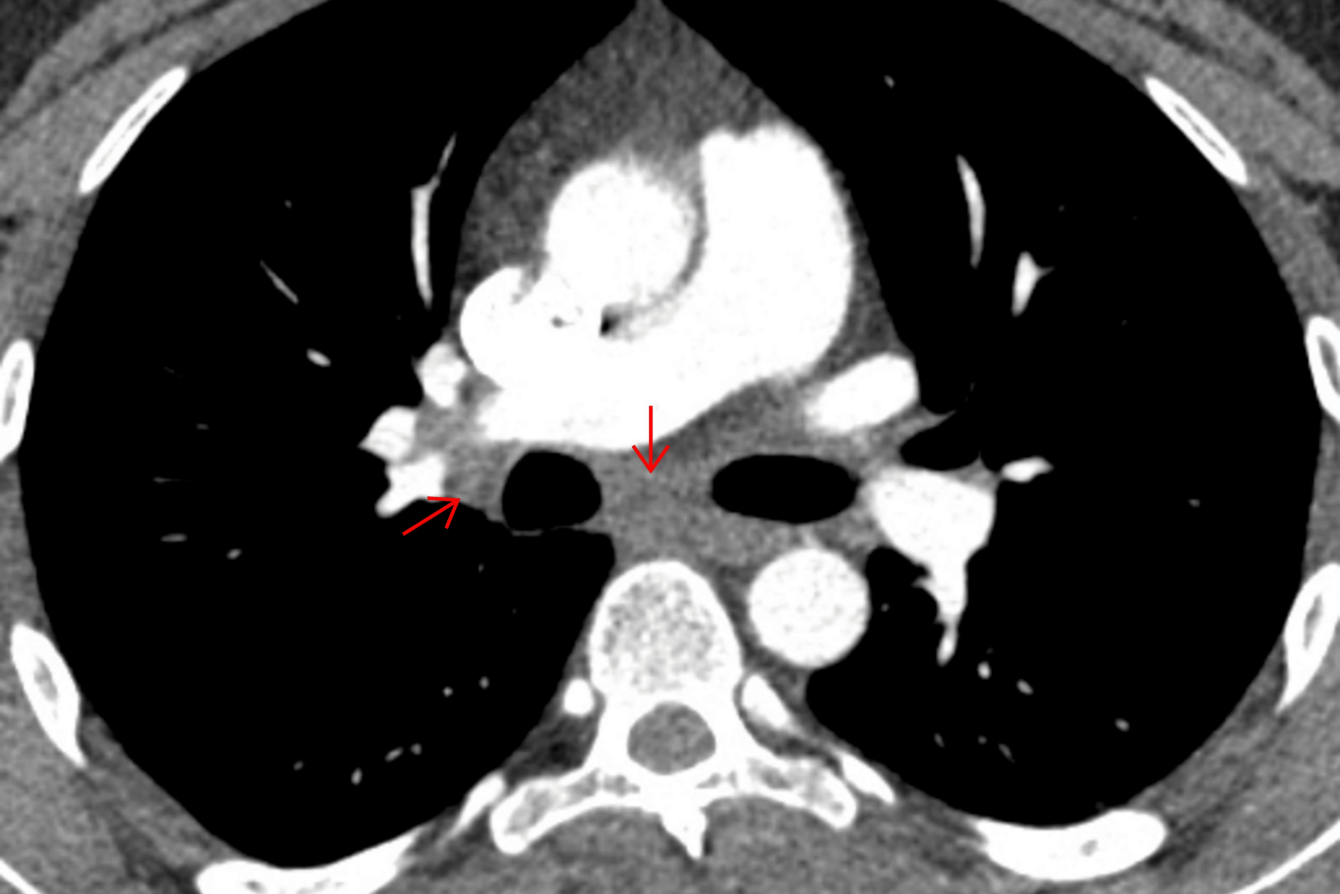
CT thorax showing complete resolution of right hilar and subcarinal lymph node CT: computed tomography

Another CT scan, six months later, showed complete resolution of all previously noted lymphadenopathy. This confirmed the transient, self-limiting nature of the disease, and the patient was discharged from further follow-up, having made a full recovery (Figures 6-7).

## Discussion

KFD, also known as histiocytic necrotizing lymphadenitis, is a rare, benign, and self-limiting condition. It is characterized by fever and lymphadenopathy, often cervical in location, and is most frequently seen in young adults, particularly from Japan, South Korea, and other Asian countries. Although it has a worldwide distribution, the actual global incidence is unknown due to underreporting and misdiagnosis [6]. The disease primarily affects young adults, particularly women, with a female-to-male ratio of approximately 4:1, and it mostly affects individuals between 20 and 30 years of age [3].

Several infectious agents, including Epstein-Barr virus (EBV), cytomegalovirus (CMV), varicella-zoster virus, human herpesviruses 6, 7, and 8, parvovirus B19, paramyxovirus, parainfluenza virus, and human T-lymphotropic virus type 1, have been suggested as possible triggers, though no definitive pathogen has been identified [7]. Autoimmune etiologies, such as an association with SLE, have also been observed [4].

The most frequent symptoms of KFD include prolonged fever, lymphadenopathy, and general malaise, all of which were present in this patient with acute or subacute symptoms onset [8]. All patients have evidence of lymphadenopathy that is mostly in the posterior cervical chain, with under 22% of cases with generalized lymphadenopathy [7,9]. The presentation of KFD with isolated mediastinal lymphadenopathy is exceedingly rare, with only two reported cases according to one study [10]. This case underscores the importance of considering KFD in the differential diagnosis of unexplained mediastinal lymphadenopathy, particularly in patients from endemic regions or with an appropriate demographic profile. Less commonly, KFD can present with extranodal symptoms such as skin lesions, which may occur in up to 40% of cases [11]. In this report, the patient had mucocutaneous ulcers, a rare symptom in KFD, which added to the diagnostic complexity.

Previous literature has emphasized the rarity of this presentation, noting that isolated mediastinal involvement can be easily confused with more sinister conditions like lymphoma or sarcoidosis. Therefore, clinicians should maintain a high index of suspicion for KFD, even in atypical presentations. The importance of KFD lies in its potential to mimic other serious conditions such as lymphoma, tuberculosis, or autoimmune diseases, making an accurate diagnosis essential for appropriate management.

The diagnosis of KFD is challenging due to its nonspecific clinical and radiological features, which often overlap with other serious conditions such as lymphoma, tuberculosis, and autoimmune diseases. Diagnosis is based on the histopathologic features obtained from excisional biopsy or FNA cytology, which is a valuable tool for the diagnosis of KFD as it is minimally invasive and allows for the examination of lymph node architecture. In KFD, FNA typically reveals areas of necrosis with histiocytic infiltrates, often with the absence of neutrophils, which helps differentiate it from other causes of lymphadenopathy, particularly infections or malignancy [5].

Multiple studies have demonstrated the diagnostic accuracy of FNA in identifying KFD, highlighting its importance in avoiding unnecessary interventions such as excisional biopsies or aggressive treatments [12].

KFD typically follows a self-limiting course, with spontaneous resolution of symptoms and lymphadenopathy within one to four months. In this case, the patient showed significant clinical improvement following initial treatment and follow-up imaging demonstrated complete resolution of mediastinal lymphadenopathy. Follow-up is essential to confirm disease resolution and to rule out the possibility of recurrence, which, although rare, has been reported in up to 3% to 4% of cases [6]. In cases of recurrence, long-term monitoring may be required to assess for potential progression to autoimmune diseases, particularly SLE, which has been associated with KFD [13].

In this case, a young male patient of Asian origin presented with fever, mucocutaneous ulcers, and an incidental finding of mediastinal lymphadenopathy, raising suspicion for tuberculous lymphadenitis. This was subsequently ruled out due to the absence of respiratory symptoms, normal lung parenchyma on CT imaging, and a negative QuantiFERON-TB Gold test. Extensive evaluation also excluded infectious causes such as EBV and CMV, as well as autoimmune conditions like SLE. FNA of the mediastinal lymph nodes was negative for malignancy. The patient’s symptoms fully resolved, with complete resolution of lymphadenopathy. This case underscores the importance of combining imaging, laboratory investigations, and FNA cytology to accurately exclude other diseases and prevent misdiagnosis.

## Conclusions

This case highlights the rare presentation of KFD as isolated mediastinal lymphadenopathy in a young male patient, a condition that typically presents with cervical lymphadenopathy. KFD, although self-limiting, can easily be mistaken for more serious conditions such as tuberculosis or malignancy due to its nonspecific symptoms. The use of FNA cytology was pivotal in ruling out malignancy and confirming KFD, emphasizing the importance of FNA in the diagnostic workup for unexplained lymphadenopathy. Awareness of KFD, particularly in its atypical presentations, is crucial to avoid unnecessary interventions. Given its association with autoimmune diseases, particularly SLE, long-term follow-up is recommended to monitor for recurrence or the emergence of autoimmune conditions. This case underscores the necessity for a multidisciplinary approach in diagnosing and managing KFD to ensure accurate diagnosis and prevent overtreatment.
